# Qualitative process evaluation of a psycho-educational intervention targeted at people diagnosed with schizophrenia and their primary caregivers in Jordan

**DOI:** 10.1186/s12888-017-1225-2

**Published:** 2017-02-13

**Authors:** Abd Al-HadiHasan, Patrick Callaghan, Joanne S. Lymn

**Affiliations:** 1 Assistant Professor in Mental Health Nursing, Nursing Department, Fakeeh College for Medical Sciences, Jeddah, Kingdom of Saudi Arabia; 2 School of Health Sciences, University of Nottingham, Queen’s Medical Centre, Nottingham, NG7 2UH UK

**Keywords:** Psycho-education Intervention, Qualitative, Thematic analysis, Schizophrenia

## Abstract

**Background:**

Schizophrenia is a serious form of mental illness that often requires long term care. Empirical findings indicate that combining a psycho-educational intervention (PEI) with neuroleptic medication to treat schizophrenia is effective. However, there is little information on the therapeutic mechanism of PEIs.

**Methods:**

A qualitative process evaluation was conducted with a purposive sample of people diagnosed with schizophrenia (PDwS, *n* = 8) and their Primary Caregivers (PCs, *n* = 9) who had received PEI as a part of an exploratory randomized controlled trial. Semi-structured interviews were conducted to explore potential processes underpinning any observed effect. Thematic analysis was used to analyze and identify prominent patterns in the data. Interviews were conducted between April 2013 and August 2013.

**Results:**

Three themes emerged from the qualitative interviews, ‘Awareness of schizophrenia’, ‘Positive impact on health and wellbeing’, ‘empowerment and enhanced confidence’, which described the variety of experiences with the intervention, although most reported that the intervention was acceptable and valued.

**Conclusion:**

This study identified that individual understanding varied between PDwS and PCs and led to differences in the ways that they used knowledge gained from the PEI in everyday situations. These data support the importance of improving understanding of schizophrenia by PDwS and their PCs to enable them to benefit more fully from medication.

**Trial registration:**

Current Controlled Trials ISRCTN78084871. Retrospectively registered 28 December 2015.

## Background

Worldwide, schizophrenia is a serious form of mental illness that strikes people in adolescence or early adulthood [1] and is listed as the eighth leading cause of disability-adjusted life years (DALY) in people aged 15–44 years. It is estimated that the economic cost of treating schizophrenia totaled approximately €93.9 billion across Europe in 2010 [2]. Interventions which reduce the morbidity and mortality burden associated with schizophrenia are therefore important and have received critical attention [3].

Data derived from a recent World Health Organisation (WHO) report, suggests that 305 individuals per 100,000 of the Jordanian population suffer from mental illness, this equates to approximately 18,300 individuals, of whom around half have a diagnosis of schizophrenia [4]. In addition, mental health services in Jordan are not well prepared to provide basic care for mentally ill people compared to developed countries. For instance, the number of psychiatric beds in Jordan is limited to around 8.27 beds per 100,000 of the population. Mental health professionals in Jordan face high workloads, with as many as 150–200 service users visiting clinics daily [4]. In Middle Eastern countries, including Jordan, the number of people who live with schizophrenia is about 3.5 million [5]. The lack of specific documentation reflects the basic infrastructure of the mental health system, which remains nascent, as well as the failure of mental health services to undertake appropriate documentation or conduct non-pharmacological specialised care [4].

Family interventions have been developed in response to the fundamental part that families play in supporting relatives diagnosed with Schizophrenia; 50%-80% of PDwS live or have daily contact with their families [6]. The theoretical rationale for these family interventions stems from previous studies showing that PDwS who live with families that have high emotional expression (EE) are more likely to relapse than those who live in low EE families [7, 8]. For instance, when the family caregivers expressed a high EE (hostility, emotional over involvement and critical comments) towards their mentally ill relative, it led to a higher relapse rate [9, 10]. Reviews of family intervention studies targeted at PDwS and their primary caregivers have consistently demonstrated positive impact on PDwS’ knowledge of schizophrenia, social, and functional recovery [8]. Psychoeducational interventions are the commonest approaches with families in developed countries [11]. The PEI model focusses on teaching PDwS and their PCs about schizophrenia, treatment, medication and managing side effects, creating low environmental stress through problem-solving strategies and coping mechanisms for illness symptoms and disruptive behaviour. Further information about the PEI used can is published elsewhere [12].

Whilst PEIs have used a variety of formats including face-to-face lectures and seminars and online, a less intrusive and more cost-effective method of providing psycho-education interventions to PDwS and their primary caregivers using a booklet format was designed for use in Jordan. The rationale for this choice was based on the practicalities of delivering this type of intervention in the wider healthcare context in Jordan. Financial constraints prevented the current study paying costs for participants to travel to sessions, as has occurred in some previous studies [10, 11], moreover provision of these costs would not be supported in an ongoing manner following study completion. In addition, and perhaps more importantly, low engagement, and high attrition rates have been seen with studies that have employed face-to-face formats [12, 13]. There is significant social stigma towards mentally ill people and their families in Arab ‘cultures’ which could deter participants from attending PEI in a face-to-face format [14, 15]. The lack of consistent and reliable internet connections also made the use of online delivery of material impractical. As a result, the research team adopted the booklets supported by follow-up phone calls to disseminate PEI, this format was considered to be more consistent and less demanding and intrusive in terms of participants’ lifestyles thus, overcoming the barriers previously associated with didactic or online formats.

Beliefs about the aetiology of mental illnesses vary across communities and cultures worldwide; some view schizophrenia as a character weakness and as laziness or punishment for not respecting ancestors [16–18], whereas others attribute mental illness to sudden fright, possession of evil spirits, use of magic, head accidents, bad genes, emotional trauma, punishment from God, or the ‘evil eye’ [19, 20]. Such beliefs about the causes of mental illness result in social stigma with the view that mental illness is the result of a person’s weak faith [21] or is punishment for one’s sins [22]. This stigmatisation of mental illness is often associated with a reduction in those affected seeking treatment from a mental health service, engaging in rehabilitation interventions, and adhering to medication [23, 24]. Similarly, young people with mental illnesses are often reluctant to seek treatment due to the fear that self-disclosure may be perceived as an individual weakness or may bring shame to one’s family [25]. In addition, in Arab countries, the distinction between physical and psychological health is not common, and has not been as historically prevalent as in so-called Western Countries. Arab-Muslim literature reveals that Arab-Muslims do not distinguish emotional or psychological distress from physical illness, and a majority of the population tend to somatise their illness, presenting physical symptoms to express an emotional disorder [19, 26, 27]. Consequently, treatments for mental illnesses are often sought from traditional and spiritual healers to the exclusion of professional mental health services [21], as they are perceived as less stigmatising and are widely considered suitable by clients. This finding is consistent with a qualitative study of Iranian families to explore their lived experience about mental illness showing family relatives’ understanding of mental illness was influenced by myths and used traditional methods as the first line of treatment [13].

Data from our recent RCT demonstrated that PDwS who received PEIs and treatment as usual (TAU) had better knowledge of schizophrenia, fewer psychiatric symptoms and fewer relapses compared with matched controls receiving TAU. In addition, PCs who received PEI and TAU reported better knowledge of schizophrenia, reduced burden of care and improved quality of life than PCs receiving TAU. The positive effects of PEI were maintained post-intervention and at 3 months follow-up in PDwS and PCs [28]. These findings are consistent with those from Iran [14], but this study used a randomized controlled trial solely and how the PEI influenced participants outcomes could not be established.

The RCT method is considered the gold standard to evaluate the effectiveness of the intervention (independent variable) on participants’ outcomes (dependent variable) through dealing with bias [29]. Whilst the RCT is considered a powerful method to provide a picture about the effectiveness of an intervention, it cannot provide an understanding of why particular responses are given. Using process evaluation however, allows the researcher to reflect on data collection, to add a deeper understanding of the phenomena under study and to determine the processes that may mediate any observed intervention effects [30]. The UK Medical Research Council guidelines for developing and evaluating complex interventions, such as RCTs, recommend integrating process evaluations alongside outcome data to maximise the interpretation of the outcomes [30]. Process evaluations can allow for the examination of participants’ views on the intervention, investigation into any contextual factors and determination of processes that may mediate any observed intervention effects [30, 31]. The use of qualitative methods have been recommended to explore and understand peoples’ experiences and perceptions of healthcare interventions in order to understand more about the dynamics of participation. This study reports the results of a process evaluation which used semi structured interviews to explore the acceptability and processes underlying any observed effect of the PEI.

Previous data examining participants’ experiences of PEI, is scarce. One previous study, reported improvements in knowledge of schizophrenia and in family caregivers’ attitudes towards their relative’s illness [32] but did not include PDwS in the qualitative component of the study. Data collection in this study was through focus groups rather than by interview, which may have inhibited family caregivers' ability to talk freely about sensitive issues in front of other participants. The only published work conducted with PDwS, emphasised the importance of the nature of the information received in the PEI but did not examine potential processes [33]. This study provides the first comprehensive investigation of the impact, and potential mechanisms underlying this impact of PEI. The study addressed the research question “How do participants (PDwS and PCs) experience the delivery of PEI?”.

## Methods

### Study design

A qualitative process evaluation was undertaken, using audio-recorded face-to-face semi-structured interviews, with PDwS and primary caregivers. This research approach is best suited to achieve a deep understanding of experiences and views from the perspective of the patients who received PEI. In this study the researchers were interested in a descriptive evaluation of our intervention (PEI) based on participant’s views and experience. Although the methodological theories (i.e. phenomenology, grounded theory or ethnography) are useful and productive in generating new insights to social behaviour [14], some authors argued that there is no need to sign up to a particular method in order to do qualitative research [15, 16]. Therefore, we used a pragmatic approach in this study. By not adopting a methodological theory in the current study, the authors tried to focus on answering the research questions, and explaining the quantitative data without the restrictions of a particular theory. Our approach had the capacity to clarify and explain the trial findings.

### Participants & procedure

The study was approved by the University Of Nottingham Faculty Of Medicine and Health Sciences Research Ethics Committee (Ref SNMP 12072012) and the Scientific Research Ethics Committee of the Ministry of Health, Jordan (Ref 9067). Written consent was obtained from all participants. Participants were PDwS and their PCs who had received the psycho-educational intervention, in addition to treatment as usual (TAU), as part of the RCT. TAU included medication, and laboratory investigations delivered by the mental health team. The intervention group participants received six psycho-educational booklets, one each fortnight for 12 weeks. Booklets, provided in sealed envelopes, were handed to participants by a member of the nursing staff at the end of the clinic appointment. Follow-up phone calls to primary caregivers were also made to ensure that they had read and understood the booklet and to allow them to ask questions about its content. Follow-up phone calls were made by one of the authors (AH) who, whilst a trained mental health nurse, was not a member of the healthcare team. The psychoeducational intervention was based on the framework of Atkinson and Coia [34]. This model was chosen as it is widely used in the literature as well as it covers Blooms taxonomy of learning domains (cognitive, affective and psychomotor). In this sense, previous reports demonstrated that a comprehensive PEI model encompassing three domains of learning is more likely to show positive influence on clinical outcomes (i.e. relapse) besides knowledge. Furthermore, the nature of this study was to investigate the use of a PEI in the community context and to provide for the different needs of both PDwS and PCs. The PEI was therefore constructed to not only provide greater knowledge of the condition and its pharmacological management but also to provide practical coping and stress management techniques which can be utilised within the home setting (Table 1).

**Table 1:** The goals and contents of PEI

Booklet Number	Goals	Contents
One	To understand the nature of schizophrenia and its symptoms.	- Diagnosis of schizophrenia according to DSM-IV. - Truths and myths about schizophrenia. - Symptoms of schizophrenia.
Two	To understand the causes of schizophrenia and the importance of the family in supporting affected individuals.	- Causes of schizophrenia - Stress vulnerability model - Role of the family.
Three	To improve participants understanding of antipsychotic medications and improve medication compliance.	- Side effects of medications. - Mechanism of action of medications.
Four	To review relapse triggers & warning signs and improve participants' ability to recognise these.	- Early warning signs of relapse. - Common relapse triggers. - Relapse management strategies. - Burden of care.
Five	To determine common problem sources in the home. To initiate techniques to solve problems.	- Problem-solving interventions in schizophrenia. - Practical advice for problem-solving.
Six	To identify stress triggers and improve stress management techniques.	- Stress management skills and strategies.

### Sample

The required sample size in qualitative research relies on researchers’ judgment and experience in examining collected data against the research purpose, method of research and sampling strategy [17], bearing in mind that qualitative researchers are more concerned about the quality of information obtained from participants than its quantity [18]. The required sample size in qualitative research relies on researchers’ judgment and experience in examining collected data against the research purpose, method of research and sampling strategy [17]. The total sample size for the RCT was 121 pairs of PDwS and their PCs with 58 pairs being allocated to the intervention arm and 63 to TAU. The sample for the qualitative component of the study consisted of eight PDwS and nine PCs selected from participants allocated to the intervention arm of the RCT. Sampling was maximum variant purposive in nature to maximise variation among interviewees according to the main socio-demographic characteristics such as gender, illness duration, educational level and the PCs relationship to the PDwS. (Table 2). Not all PDwS and/or PCs originally selected consented to be interviewed. Purposive sampling then continued until representation from across the majority of demographic characteristics was achieved. Reluctance to participate in interviews is not uncommon in the context of such families, who often believe that excess emotion, such as anger, endangers health and therefore should be controlled. There may also be a reluctance to reveal private thoughts and feelings in front of others, especially strangers [19]. In the beginning, the decision about this sample size was flexible. However, it was guided by achieving variation in the socio-demographic background of selected participants and was influenced by data saturation. The inclusion criteria in the study were the participants had to receive the full PEI and complete outcome measures at baseline and follow-up participate voluntarily.

**Table 2:** Characteristics of interview participants compared to trial participants

Characteristic	Trial sample - number		Interview sample – number (% of trial sample demographic)	
	PDwS	PC	PDwS	PC
Participants	58	58	8 (14%)	9 (16%)
Gender:				
Male	38	10	6 (16%)	0 (0%)
Female	20	48	2 (10%)	9 (19%)
Age (years):				
≤ 20	2	0	0 (0)	0 (0)
21-30	7	2	2 (28)	1 (50)
31-40	25	16	3 (12)	3 (19)
41-50	19	21	1 (5)	4 (19)
≥ 51	5	19	2 (40)	1 (5)
Education Level:				
Primary School or below	18	15	2 (11)	2 (12)
Secondary School	22	14	2 (9)	3 (21)
College or above	18	29	4 (22)	4 (14)
Diagnosis:				
Schizophrenia	32	n/a	4 (13)	n/a
Schizoaffective disorder	26	n.a	4 (15)	n/a
Illness duration (years)				
≤ 2	6	n/a	2 (33)	n/a
3-5	12	n/a	2 (17)	n/a
≥ 5	40	n/a	4 (10)	n/a
Relationship to PDwS:				
Parent	n/a	24	n/a	1 (4)
Sibling	n/a	10	n/a	3 (30)
Spouse	n/a	20	n/a	5 (25)
Child	n/a	4	n/a	0 (0)

### Data collection

Semi-structured interviews were the data collection method. A topic guide, developed by the authors, prior to the start of the main RCT, was used to guide the interview process in order to increase the breadth and depth of information obtained from the participants. The topic guide was devised based on the study aims which focused on participants’ experiences of the PEI and how it may have impacted on their life, as described in Table 3 [16].

**Table 3:** Interview schedule

1. Background
• Welcome and thank participant
• Introduce interview topics
2. Disease History
• How many years ago were you/ your relative diagnosed with schizophrenia?
• How long had you / your relative been ill prior to diagnosis
• PCs – how did you find out about your relatives treatment and care?
• How did you feel when you/your relative was diagnosed with schizophrenia?
3. Impact on social life
a. How did your / your relatives disease impact on you / your life (social, working and economics)?
b. PCs – how much support have you had in caring for your relative
4. Impact of education intervention?
a. Did you read the information booklets
b. If no – why didn’t you read the booklets
c. If yes – did you find them useful – has your knowledge level improved
d. If yes (PDwS) - do you feel that the increased knowledge of the disease has helped you manage your disease symptoms
e. If yes (PCs) - do you feel that the increased knowledge of the disease has helped you cope better with the burden of caring
5. Information needs
a. What was the most useful component of the educational booklets
b. What was the least useful component
c. Are there any other aspects of the disease which you would have liked to have been included
d. How did you feel about the timing of the intervention with education booklets being made available every fortnight
6. Format of education delivery
1. How did you feel about the use of booklets to deliver this knowledge?
a. Is there any other delivery format that would have been easier/better for you/your PC?
7. Experience of study
1. Why did you take a part in this study?
2. What are you expectation when did you participate in this study?
3. Has taking part in this study altered your view about schizophrenia? Caring for relatives with schizophrenia? If so please explain?
4. Would you recommend this educational intervention to other patients/carers? Why?

The purpose of the interviews was to explore the acceptability of the intervention and examine potential processes underlying the observed effects from the RCT [20]. Interviews were conducted by one of the authors (AH), a trained interviewer, at a time and a location suitable to each participant. The majority of these interviews were completed in a private room in the mental health clinic; however two interviews were conducted at the participants’ homes, at their request. Four interviewees were pairs of PDwS and their PCs. However, these interviews took place separately in order to ensure their responses were not influenced by each other. No time limit was imposed on the interviews, but the average time taken for most interviews was between 45–60 min. Before commencing interviews, participants were reminded that they could ask any questions or refuse to answer any question. In addition, they were informed about their right to stop or withdraw from interviews at any time without a reason. All participants were interviewed once and the interview was audio-recorded, enabling the interviewer to be more focused and interactive with interviewees in order to follow the issues raised, to ask for clarification on some issues and to keep the interviews focused on the research phenomena. Audio recording also enabled exact transcription of respondents’ narratives, avoiding the problems of recall and note-taking.

All interviews were carried out in Arabic, transcribed in Arabic and translated into English for purposes of analysis. Transcripts were then back-translated into Arabic to ensure that no loss of meaning of participants’ experience had occurred, and to validate the conceptual equivalence of translation [35]. English version transcripts were read by both AH and JL. AH conducted the initial coding for all interviews, JL performed initial coding for a random selection of interviews and coding themes derived from the interviews were compared.

### Data analysis

All interviews were analyzed manually using thematic analysis [21] in six stages:

1. Interview transcripts were read and re-read repeatedly in order to obtain a broad understanding of the participants’ views [18].
2. Initial codes were generated; a complete coding approach was utilized in order to identify anything and everything across the data set which might have relevance to the research question [21, 22].
3. All similar codes or meanings were collated together into potential themes. Relevant extracts from the data set were collated to form themes [21, 22]
4. Potential themes were reviewed and a thematic map generated.
5. Identified themes and subthemes were checked against each other and the dataset to ensure they were coherent, distinctive, consistent and working together.
6. Themes were reflected at the semantic level of data with illustrative quotes from participants selected [21].

### Rigour

The maximum variant purposeful sampling used in this study ensured that all sub-groups within the research setting were given a voice so that comparisons could be used to construct commonalities and differences in interpretation across groups. The researcher involved in interviewing participants had previously been engaged with participants in terms of conducting baseline measures and making follow-up phone calls to check receipt of the PEI. This researcher was not involved in the collection of follow-up data so had no knowledge of the impact of the PEI on individual PDwS or PC outcome measures. Similarly, all interviews were conducted prior to the analysis of the quantitative data generated in the RCT, using a schedule designed at the outset of the study, to explore the experience of the PEI on PDwS and their PC without being influenced by knowledge of the impact of the PEI on outcome measures.

Source triangulation involved the research team constantly comparing the data that emerged from participants’ interviews and presenting quotations from participants [23]. One of the researchers (JL), familiar with qualitative research methodologies acted as a peer to achieve credibility. The study team adhered to the Consolidated Criteria for Reporting Qualitative research (COREQ), which aims to promote complete and transparent reporting, and improve the rigor as well as dependability of qualitative research (Table 4).

**Table 4:** Consolidated criteria for reporting qualitative studies: 62 items checklist

No. Item	Guide questions/description	Considered
Domain 1: Research team and reflexivity		
Personal Characteristics		
1. Inter viewer/facilitator	Which author/s conducted the interview or focus group?	Yes
2. Credentials	What were the researcher’s credentials? e.g. PhD, MD	Yes
3. Occupation	What was their occupation at the time of the study?	Yes
4. Gender	Was the researcher male or female?	Yes
5. Experience and training	What experience or training did the researcher have?	Yes
Relationship with participants		
6. Relationship established	Was a relationship established prior to study commencement?	Yes
7. Participant knowledge of the interviewer	What did the participants know about the researcher? e.g. personal goals, reasons for doing the research	Yes
8. Interviewer characteristics	What characteristics were reported about the inter viewer/facilitator? e.g. Bias, assumptions, reasons and interests in the research topic	Yes
Domain 2: study design		
Theoretical framework		
9. Methodological orientation and Theory	What methodological orientation was stated to underpin the study? e.g. grounded theory, discourse analysis, ethnography, phenomenology, content analysis	Yes
Participant selection		
10. Sampling	How were participants selected? e.g. purposive, convenience, consecutive, snowball	Yes
11. Method of approach	How were participants approached? e.g. face-to-face, telephone, mail, email	Yes
12. Sample size	How many participants were in the study?	Yes
13. Non-participation	How many people refused to participate or dropped out? Reasons?	No
Setting		
14. Setting of data collection	Where was the data collected? e.g. home, clinic, workplace	Yes
15. Presence of non-participants	Was anyone else present besides the participants and researchers?	Yes
16. Description of sample	What are the important characteristics of the sample? e.g. demographic data, date	Yes
Data collection		
17. Interview guide	Were questions, prompts, guides provided by the authors? Was it pilot tested?	Methods
18. Repeat interviews	Were repeat inter views carried out? If yes, how many?	N/A
19. Audio/visual recording	Did the research use audio or visual recording to collect the data?	Yes
20. Field notes	Were field notes made during and/or after the interview or focus group?	Yes
21. Duration	What was the duration of the inter views or focus group?	Yes
22. Data saturation	Was data saturation discussed?	Yes
23. Transcripts returned	Were transcripts returned to participants for comment and/or correction?	No
Domain 3: analysis and findings		
Data analysis		
24. Number of data coders	How many data coders coded the data?	No
25. Description of the coding tree	Did authors provide a description of the coding tree?	No
26. Derivation of themes	Were themes identified in advance or derived from the data?	Yes
27. Software	What software, if applicable, was used to manage the data?	No
28. Participant checking	Did participants provide feedback on the findings?	No
Reporting		
29. Quotations presented	Were participant quotations presented to illustrate the themes/findings? Was each quotation identified? e.g. participant number	Yes
30. Data and findings consistent	Was there consistency between the data presented and the findings?	Yes
31. Clarity of major themes	Were major themes clearly presented in the findings?	Yes
32. Clarity of minor themes	Is there a description of diverse cases or discussion of minor themes?	Yes

The domains of this checklist (research team and reflexivity, study design, and data analysis and reporting) provide a transparency of research methods that allow readers to assess the trustworthiness and transferability of the findings of the primary study to their setting [24].

## Results

### Sample description

The mean age for PDwS was 31.8 years and for PC was 48 years. The majority of participants were female primary caregivers and male PDwS. More than half of interviewees had attained secondary levels of education or higher. The majority of primary caregivers were female and more than half of PDwS had been diagnosed with schizophrenia for fewer than five years.

### Interview data

**Fig. 1:**
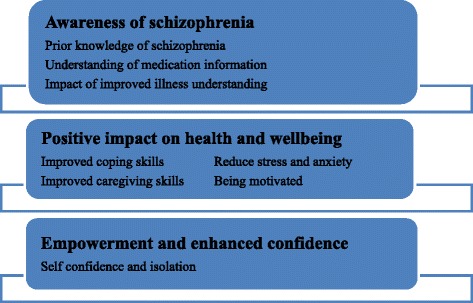
Themes and Subthemes Emerged from People Diagnosed with Schizophrenia and Primary Caregivers Interviews

Three overarching themes emerged from the interviews and are described in Fig. 1.

### Awareness of schizophrenia

This theme describes the impact the PEI on enhanced illness cognition and captures the effect of this enhanced understanding on the ability of PDwS and their PCs to recognise and regulate clinical symptom expression.

#### Prior knowledge of schizophrenia

Interview data demonstrated that almost all participants had a limited knowledge of schizophrenia, regardless of their age and educational attainment. This was evidenced by the fact that more than two-thirds of the participants who were interviewed believed that schizophrenic patients had two personalities and that their interactions with others relied on the predominant personality (aggressive or calm):

*“….. I thought that my illness was considered as many personalities inside my body […] especially when I heard voices…..” (PDwS7)*

*“….. We believed that [schizophrenic] patients had multiple personalities…..” (PC4, Wife)*

Similarly, PDwS often drew attention to a wide range of views about the nature of their illness expressing the notion that schizophrenia was caused by black magic or evil spirits.

*“….. In addition to that, we believed these symptoms were regulated by other bodies that lived inside my body or that it was related to sorcery….” (PDwS5)*

Primary caregivers expressed overwhelming feelings of guilt over their relative’s diagnosis with schizophrenia, believing that this was a result of a direct failing on the part of the family:

*“….. Furthermore, I have {ah, ah…..} believed the cause of her illness to be due to a mother’s neglect, because I {ah} had worked outside the home for a long time previously…..” (PC6, Parent)*

In most of the interviews, the introduction of the PEI resulted in an inconsistency between interviewees’ prior thoughts of the causes of schizophrenia and the scientific explanation for the illness. More than half of the interviewees reported that the PEI provided information that helped develop their knowledge of the potential causes of schizophrenia and its impact on their relatives:

*“….. When I read the booklet, I [PC] felt ashamed and cried […] as the meaning of [schizophrenia] had changed for me. The booklets said that schizophrenia does not impact on personality and is connected with cognition and emotion…..” (PC1, Wife)*

Participants attributed poor knowledge and understanding of their illness for several reasons, including the paucity of educational resources for mentally ill people, and the stigma attached to mental illness in Jordan:

*"….. Mental illness is looked down upon. I can’t tell others I have a mental illness or I can’t visit a psychiatrist because he is associated with crazy people and it is said that he has lost his mind….." (PDwS1)*

*“….. It is a difficult topic to talk about in our culture. We live in the village area where people are not educated …..” (PC3, Sister)*

#### Understanding of medication information

Primary caregivers commonly reported a negative attitude towards medication use. It was noted that this had changed due to the information they had obtained from the PEI. This related to the acknowledged importance of continuing medication use to prevent relapse.

*“….. The booklet says that stopping the medication without informing the physician will lead to serious [relapse], and it’s so hard to return a patient’s status to baseline […..]……so I changed this bad habit, and I became more adherent with medications and never tried to stop the medication since I read this information…..” (PC1, Wife)*

Similarly, some primary caregivers described that they had previously thought that some of the medication side effects were actually deliberate behaviours exhibited by their relatives. For instance, two primary caregivers said that:

*“….. It is important to know about medication [….]. I knew the side effects of the medication, but I believed these side effects […] such as fatigue and muscle spasm were intended behaviour to just sleep….” (PC8, Wife)*

Interestingly, having knowledge about antipsychotic medication was found to influence the primary caregivers in different ways. Some interviewees commented that having this information about medication was not of great benefit to them:

*“….. I think it’s more important for me to know about living and caring with [schizophrenia] than its medication […] my knowledge about his illness is very limited…..” (PC9, Wife)*

Conversely, improved understanding of medication information allowed some primary caregivers to feel that they were an active part of the treatment process in determining the best medication for their relative:

*“….. If I observed that the medication is not suitable for him and the illness symptoms such as hearing voices or talking to no one have returned, I can return back to the physician to review this medicine…..” (PC4, Wife)*

In the context of medication supervision skills, some primary caregivers reported taking a more active role in ensuring medication compliance:

*“….. Sometimes he takes a tablet, but he moves it out when I leave. Now I stay until I am sure that he chewed and/or swallowed the tablet. Previously we had a problem when he used to forget to take his medicine …..” (PC4, Wife)*

However, several PDwS reported a change in attitude towards medication following the PEI. Close investigation of the interviews of PDwS revealed that different mechanisms were used among interviewees to remind them to take medications on time, including putting medication in obvious places, taking medicine at the same time daily, or depending on the primary caregivers to remind them:

*“….. Previously, my family placed the tablets in my food or juice* [25]*, because I refused to take them […]. However, now I take my medication on time [….]. I put my medication in the dining room to take it right after dinner" (PDwS7)*

A key conclusion drawn from the interviews suggests that the PEI contributed effectively to improving their awareness of the importance of medication and changed their attitudes towards it. Therefore, they became more empowered to actively engage in the treatment regimen.

#### Impact of improved illness understanding

Another key knowledge component that participants reflected upon was the acquisition of information about relapse warning signs. Indeed, almost all the participants post-intervention reported an ability to detect early warning signs of relapse through observing the changes in their relative’s (PDwS) behaviour over time.

*“…..One of the booklets provided suitable information about [relapse]. Sometimes, I saw or felt {ah…} her change after a stabilised or calm period. When these changes occurred [….]. I knew this is a [relapse] sign and the booklets helped me handle her in this situation to avoid [a relapse]…..” (PC3, Sister)*

Similarly most of the PCs interviewed mentioned monitoring psychiatric symptoms regularly and identifying early signs of potential relapse to prevent hospitalisation of their relative as described in the quote below:

*“….. Moreover, the educational booklets informed us about [relapse] symptoms and causes, so we try to avoid these causes as much we can. When I see some symptoms appear in him such as disorganised speech, non-compliance with medicine, changes in facial expression as uncontrolled movement in the eye or irritability, excessive crying or joking we go to the physician immediately. As a result, the physician adjusts the medication dosage which might prevent hospital admission” (PC5, Sister)*

Interestingly PDwS also felt able to utilise this knowledge with a small number expressing that they were able to recognise and monitor themselves to detect signs of potential relapse signs, enabling them to take preventive action, avoiding hospitalisation as a result.

*“….. The relapse booklet discussed relapse prevention techniques, so that it is possible to differentiate between illness symptoms and relapse signs…..” (PDwS6)*

Despite the apparent benefits of the intervention, the increased knowledge and understanding of schizophrenia, particularly the potential hereditary nature of the illness prompted some concern:

*“….. When I learnt the different ways of diagnosing the illness and its causes {ah, ah…}, I figured out how I can treat these causes. For instance, when I found out that one of his illness causes was hereditary, I have become more aware for illness symptoms if they ever appear on our children. Especially since one of his brothers has the same illness.…..” (PC4, Wife)*

For PDwS, their increased knowledge of schizophrenia allowed them to understand the chronic nature of the illness and what this would mean for their lives.

*“….. Sometimes I thought I completely recovered from my illness, especially when the sounds disappeared, but the educational material said {ah…} this illness is not curable and if I stopped taking […] the required medication, I could have a relapse…..” (PDwS7)*

Similarly, some of the participants suggested that including some information about illness symptoms would be more helpful to differentiate schizophrenia symptoms from medication side effects or the symptoms of other illnesses. This is illustrated by the following quote:

*“….. Furthermore, {…ah…} I can now explain some of my symptoms and attribute them to my illness or medication or others reasons. For example, constipation is considered to be a medication side effect {ah}. Also, I did not know that sexual dysfunction was due to my illness…..” (PDwS6)*

More significantly, having gained information about schizophrenia some PDwS reported they were better able to utilise this information in real life situations. Indeed a small number of the interviewed PDwS said they were able to recognise and monitor themselves to detect any relapse signs when they appear, enabling them to take preventive action against the illness, avoiding hospitalisation as a result.

*“….. The relapse booklet discussed relapse prevention techniques, so that it is possible to differentiate between illness symptoms and relapse signs…..” (PDwS6)*

However, nearly half of the respondents (PDwS) preferred to engage their primary caregivers in the PEI because they felt they would be better placed to recognise relapse signs. This issue is illustrated by this quote from three interviewed PDwS:

*"….. I think that teaching family about these signs is better because when I relapse I cannot feel these signs and cannot use my knowledge to manage these issues….." (PDwS8)*

### Positive impact on health and wellbeing

The PEI helped increase participants’ understanding of schizophrenia, and equipped them to cope with the processes associated with daily living more effectively. The sub-themes which came out from the data were associated with improving both coping skills and caregiving skills, reduced stress and anxiety and motivation.

#### Improved coping skills

Most of the primary caregivers reported that the PEI improved their awareness of the importance of communication as a skill as well as their role in enhancing communication. For example:

*“….. But education improved some of the practical aspects in our life, as I felt some positive changes in our life [.....]. Having information about [schizophrenia] helped us to improve our communication methods with patients. I used to communicate with him in a tough and stringent way and often neglected him, but now I changed and listen to him….. ” (PC9, Wife)*

From the primary caregivers’ accounts in the interviews, two main issues were deduced in relation to the impacts of the PEI on coping skills. Firstly, several of the primary caregivers’ accounts suggested previous difficulties in communication with their relatives due to a lack of essential communication skills and an insufficient understanding of schizophrenia. However, they reported that the PEI assisted them in improving their communication skills:

*“….. I did not spend a lot of time talking to him […]. I understand now what my role is in supporting him and to make sure he also understands his role in the treatment plan, hence, our communication is improved…..” (PC9, Wife)*

Secondly, other primary caregivers, particularly spouses, highlighted the difference resulting from improving communication skills with their relative.

*“….. Improving our knowledge of his illness helped us to change our attitudes towards him and improve our communication in certain ways, so that it reduced our problem levels as well as emotionally exaggerated level” (PC2, Wife)*

Moreover, a better understanding of the nature of schizophrenia enabled them to develop strategies to cope with the illness. One-third of the primary caregivers reported how they were able to incorporate the skills learnt in managing illness symptoms in an appropriate way. This is illustrated by the following comment:

*“…..We intended to minimise the time that she spent alone and encouraged her to interact with us to reduce her isolation [….].” (PC7, Sister)*

Contrary to previous views, one primary caregiver expressed concern that applying the booklet’s contents was sometimes counter-productive. For example, in the case of the PDwS hearing voices and performing certain orders (conditions that are sometimes associated with uncomfortable feelings), the booklet advised the primary caregiver to intervene and tackle the sounds, but this could lead to serious confrontation:

*“….. Once when I spoke with him, he suddenly started to talk with himself {….}. He was very nervous and started to shout at me […]. At that moment I felt so scared that I went outside the house…..” (PC8, Wife)*

#### Improved caregiving skills

Caring for relatives diagnosed with schizophrenia can affect both the physical and psychological health of the primary caregiver. Several of the primary caregivers interviewed reported finding the caregiving process stressful, and this is reflected in some of the participants’ views:

*“….. I am always scared in caring and monitoring her, because mental illness requires specific care and preparation and is not like somatic illness…..” (PC3, Sister)*

Approximately half of the primary caregivers interviewed reported that following the PEI, they had experienced an increased awareness and recognition of specific stressors. This increased awareness often resulted in adapting their behaviours more appropriately to manage stressful situations. The following views are examples of primary caregivers adopting the new caregiving skills they developed through changing their attitudes:

*“….. When we found out the causes of her illness, we tried to avoid what we could avoid to remove these causes. For instance, when I read that stress and pressure were the main sources for mental hospital admission, we tried to minimise the stress level in the home environment …..” (PC3, Sister)*

In addition to their own caregiving skills, analysis of the narrated accounts highlighted their use of other strategies, such as seeking support from close family members:

*“….. I was saying {ah…} that I often visit my husband’s family to express to them my concerns about my husband behaviours […]. When I feel depressed or anything I go to visit them to vent my feelings. …..” (PC1, Wife)*

Additionally, one of the primary caregivers reported the use of religious practices as a caregiving skill, by asking her relative to pray to relieve his suffering. Moreover, she reflected on the importance of religious practices to keep her relative active at all times. This is illustrated by the following comment from the primary caregiver:

*“….. My mother was treated for depression. This happened to her due to lack of faith and worship, but when she prayed, all of these conditions eliminated […]. I ask him to pray so as to improve his condition and increase his activity…..” (PC9, Wife)*

Developing or adopting new caregiving skills among the primary caregivers appeared to be influenced by a strong desire of the primary caregiver to improve their life, reduce the burden of care and to reduce any negative impact on their ill relative's life. For this reason, they seemed eager to sometimes use more than one caregiving skill to avoid any difficult situations and their consequences on their relatives.

*“….. If I did not change my reactions to his behaviour {…} he might have relapsed, and it may have impacted negatively on me and my family members [….]. He might {ah} have hurt me or my children […] I meant to say that when I changed my reactions with him he was being calmer….." (PC4, Wife)*

#### Reduced stress & anxiety

A central issue that appeared prominently in the PDwS view was related to how they utilised their new knowledge and understanding in managing illness symptoms. Some PDwS indicated that the acquisition of knowledge of schizophrenia from the PEI booklets was directly employed to overcome anxiety and stress levels associated with these illness symptoms:

*"….. I felt more power when I had information about my illness to face it […]. I reminded myself I should be stronger than this to prevent its progression….." (PDwS2)*

Other participants described behavioural modification to deal with stress related to the illness, as they developed new coping skills::

*“….. As I said I was a nervous man, so I used the booklet’s suggestions in controlling stress and nervousness by doing physical exercise, listening to music and others…..” (PDwS1)*

#### Being motivated

Another important issue that was evident from the interview data was that more than half of the PDwS reflected on the positive outcomes associated with the PEI which allowed them to connect the information gained with the motivation to seek treatment and treatment compliance, as well as its contribution to life quality. The following extracts reflect this notion:

“….. *My health status now is comparatively better. Also, I started to join my carers when they go to clinic monthly” (PDwS3)*

In this sense, an interesting finding that emerged from the PDwS accounts is the view that the PEI appeared to influence the manner in which schizophrenia was perceived. For example, two interviewed participants said that:

*“….. I am optimistic and motivated to take my medication so that I have better chances of recovery and controlled my illness progression…..” (PDwS4)*

The interpretation of the data presented above suggests that, regardless of the illness duration, the PEI positively impacted PDwS awareness. This in turn altered their views of their illness and encouraged them to accept it and to be more compliant with treatment regimens, including medication and appointments. It is evident that the current treatment approach for these patients in the government clinics did not adequately meet their needs.

### Empowerment and confidence

This section describes how having schizophrenia impacts on a primary caregivers’ and PDwS social life and discusses how the PEI contributed in improving their personal self-confidence and social interaction.

#### *Self-confidence and socialisation*

Prior to their participation, most of the primary caregivers that were interviewed commented on the negative changes in their lives after having a relative diagnosed with schizophrenia. Employed primary caregivers in particular, were acutely aware of the responsibility of the caring, supporting and monitoring that they had to adopt:

*“….. […] it means {ah} I am responsible for everything; I need to manage the conditions in my home alone […]. I have to take decisions alone and I am responsible for looking after my children alone…..” (PC4, Wife)*

Additionally, some of the primary caregivers elucidated the negative impacts of schizophrenia on their social lives in a number of ways. They described the social stigma that is attached to mental illness or certain behaviours of their relatives diagnosed with schizophrenia, which led them to terminate social relationships with their friends and relatives:

*“….. Moreover, embarrassment and guilt because of her mental illness urged us to minimise our contact with others in order to reduce the number of people who knew about her illness…..” (PC3, Sister)*

Following the PEI, however, a number of primary caregivers, reported that their improved knowledge and understanding of schizophrenia increased their social interactions and promoted greater optimism for the future:

*“….. My social life was impacted as a result. I was scared that his personalities would appear in front of other people who would observe it. But when I read that the illness impacts on cognition. Hence, I became more social and visited my family and had lesser fear than before, giving me some kind of happiness now…..” (PC1, Wife)*

A few of the primary caregivers attributed the increased knowledge level of schizophrenia with the self-confidence to face the community with more courage:

*“….. Improving our understanding of illness enhanced our self-confidence that improved our moral support which allowed us to interact with others without shame or less respected for people with this disease…..” (PC5, Sister)*

Some primary caregivers indicated that the PEI restored their relationships with relatives who had hitherto become estranged due to the illness. This is because the primary caregivers’ improved understanding of the nature of the illness enabled them to absorb and prevent problems proceeding at an earlier stage so as to reduce the opportunity of exacerbation:

*“….. My participation in the study {ah} provided me with a positive picture about his illness, so my [phobia] in my interaction with him reduced. I now became more reflective in problem situations, so he did not need to hit or hurt me like previous time…..” (PC8, Wife)*

Critical examination of the interview transcripts showed that almost all the PDwS spoke of a considerable negative change in their social lives due to schizophrenia. PDwS attributed these changes to several factors, including a lack of self-confidence, negative community views, and the nature of the illness and medication side effects. For example, a number of PDwS were discouraged from being socially active as a result of these factors:

*"….. I was afraid to interact with others because I had a fear {…} of speaking any inappropriate words, or making any inappropriate action or behaviour….." (PDwS4)*

PDwS expressed a sense of frustration because of their lack of self-confidence to interact with others, also due to the negative community view of mental illness in Jordan. Overall, these PDwS appeared to be calling for the dissemination of the PEI information to the wider level in society in order to change negative community views, which may enhance socialisation for PDwS:

*"….. Because mental illness among people is taken to mean that a man is crazy and I wanted to be away from all of these negative views, I preferred to be away from others….." (*PDwS8)

It can be deduced from the analysis of the data that there is wide agreement among PDwS with this view. Most of the interviewed PDwS reported social stigma was a significant obstacle, making it difficult for them to communicate with others. They expressed that:

*"….. Many of my friends when they knew I had a mental illness, decided to discontinue our relationship {ah…} because we have a stigma in our community and they fear having some of these negative views associated with them […]…." (PDwS4)*

However, almost all of the PDwS interviewed acknowledged the positive effects of the PEI on their social lives, directly or indirectly. According to the PDwS, the PEI improved their self-confidence by allowing them to become more socially active. This notion of self-confidence was raised by most of the PDwS, albeit from different perspectives. For instance, some PDwS pointed out that improving their knowledge of schizophrenia reduced the social stress associated with public speaking:

*"….. When I knew my illness symptoms, it gave me a sense of confidence, I felt brave. I knew this social anxiety or stress was related to the illness symptoms, not anything related to my personality. ….." (PDwS8)*

Other PDwS recognised that having knowledge of schizophrenia meant that the fear and guilt they felt dissipated, allowing them more control over the illness symptoms:

*“….. I feel more confident now, and my fear when I talk with people reduces; this information {ah…} made me more confident, so I began to talk with others without fear or guilt….." (PDwS7)*

Others focused on the impact of the PEI on their self-confidence through reinforcing the notion that schizophrenia is similar to other chronic illness that can be lived with for a long time:

*“….. When I read the booklet, I knew it is a common illness and my self-confidence improved, so I am now interacting with others more than the previous time…..” (PDwS1)*

The above extracts illustrate the importance of improving the knowledge and understanding of PDwS concerning the illness, so as to eliminate or minimise the perceived stress, fear and guilt that can be associated with the illness, particularly as a result of the cultural stigma attached to mental illness within Jordan. Furthermore, these accounts together suggest that having an adequate knowledge of their illness appeared to improve their self-confidence and social life. The PDwS’ views highlighted the inevitable clash between self-confidence and social networking when a number of participants reported that being diagnosed with schizophrenia appeared to produce low self-confidence and social isolation as a result.

## Discussion

The purpose of this study was to explore the acceptability and processes underlying any observed effect of the PEI on the participants’ outcomes. The intervention was perceived as being valuable in enhancing participants’ knowledge and understanding of schizophrenia as well as promoting their well-being. The findings of this process evaluation corroborated the quantitative findings of the RCT, published elsewhere, that indicated statistically significant effects of the intervention [26].

The qualitative data illuminates how the intervention impacted participants’ knowledge and understanding and how they applied this understanding in their lives to their benefit. The limited knowledge levels of schizophrenia among participants prior to receiving the intervention seen in this study correlates well with previous literature [27–29]. These data clearly demonstrate that the limited knowledge of schizophrenia was associated with self-stigma, shameful feelings and a pre-occupation with negative psychological feelings (i.e. depression, low self-esteem), all of which have been linked with lower treatment adherence [30] and a poor quality of life [31]. The qualitative data reflects the impact of improving knowledge and understanding of illness on self-efficacy, self-esteem and empowerment. The findings also accord with those of other studies that emphasise the importance of an improvement in knowledge in enhancing PDwS and their family caregivers self-efficacy [36] and empowerment [32, 33], which may influence health outcomes [34, 37].

Additionally, the majority of participants who were interviewed explained that the information obtained from the intervention helped them to manage their condition, and that it gave them the courage to confront stress and stigma. Furthermore, in this study, empowerment modified the way participants perceived their illness, and encouraged them to take more control over negative events and to handle internalized stigma better, which may in turn have led to their improved wellbeing (i.e. being less stressed, more optimistic etc.). This finding has much support in the literature, which indicates that improving family caregivers’ understanding of the illness might have assisted them in reappraising caregiving demands and better handling of maladaptive behaviour [38].

Moreover, an understanding of the illness enabled them to know who they are, rather than simply internalise and accept societal prejudices about schizophrenia; they felt they were like other individuals who face problems as a part of daily life and used their skills to handle this. These findings are in accordance with those reported in other studies [35, 36]. In addition, the issues of problem-solving and communication skills discussed in the booklet provided practical strategies to handle challenging situations. Indeed, the themes that emerged from the data reflected the notion that knowledge is power; having an understanding of the illness helped PDwS and primary caregivers to discuss the illness and put questions to mental health professionals, as well as to engage actively in their treatment decision-making process. Commenting on this, others [39] argued that an increase in the awareness of family relatives about medication may lead to their improved medical vocabulary and enable them to ask psychiatrists questions concerning their ill relative.

The importance of knowledge improvement for these study participants lies in its influence on their ability to take an active role in their own treatment regimen, rather than merely being a passive recipient. Therefore, it is likely that the knowledge and skills obtained from the intervention provided PDwS and their primary caregivers with an understanding of the nature of the illness and treatment options, which in turn enabled them to be empowered to discuss suitable medication options with the mental health team. For instance, the interviewed primary caregivers indicated they felt sufficiently confident to discuss suitable antipsychotic medication for their ill relative with psychiatrists. Likewise, this view was also echoed in another study, which concluded that mentally ill people who are uninformed about medication and its side effects are at a higher risk for discontinuation of therapy without proper consultation from psychiatrists [40].

In the current study, primary caregivers had different strategies to handle relapse, such as controlling the stressors in the home environment or increasing medication dosages based on their psychiatric consultation. It should be noted that experts in schizophrenia research estimate that the time interval between early signs and relapse is about one week [41]. Therefore, applying a suitable counter-measure during this interval (one week) could prevent relapse with hospitalisation by increasing medication doses until the precipitating factors have been addressed and resolved [42].

With respect to the relapse prevention, the content of the PEI included in a booklet about the early signs of relapse which may have contributed to the reduction in relapse with hospitalisation. This might have allowed participants to take immediate action in case these symptoms reoccurred. Most of the interviewed primary caregivers expressed their improved ability in monitoring the early signs of relapse and taking suitable actions in order to forestall or prevent relapse progression towards hospitalisation. Another study demonstrated the importance of the ill relative’s close observation for an effective monitoring of early warning signs of relapse [43].

As a corollary, it is therefore possible that knowledge gained from the intervention enabled and empowered study participants to identify environmental triggers of relapse, and to implement relapse prevention strategies earlier rather than later or not at all. In addition, better adherence with medication and adjusting doses with psychiatrists may explain relapse results. Similarly, it was noted this finding was corroborated with other study finding [44], which showed that enlisting primary caregivers who had frequent contact with their mentally ill relatives in the PEI enabled them to detect prodromal signs of relapse and helped in managing stressful situations, as well as supporting medication adherence more than other family relatives.

The notable finding from this study is that most of the interviewed PDwS explicitly linked the improved knowledge of schizophrenia gained from the PEI with the perceived improvements in their coping strategies, especially with regard to the debilitating symptoms of schizophrenia (e.g. hallucinations). This helped them cope with these symptoms in their lives, resulting in improved mental conditions. For instance, some interviewed PDwS utilised a cognitive approach to cope with hallucinations. This is supported by [45], which indicated that teaching PDwS to cope with the symptoms of schizophrenia may enable them to relabel them as harmless symptoms of their illness, or to restructure paranoid thought content. The findings of this study are in line with those of [46], which showed that cognitive, behavioural and physiological strategies were commonly cited among Taiwanese PDwS as ways to cope with auditory hallucinations

**Fig. 2:**
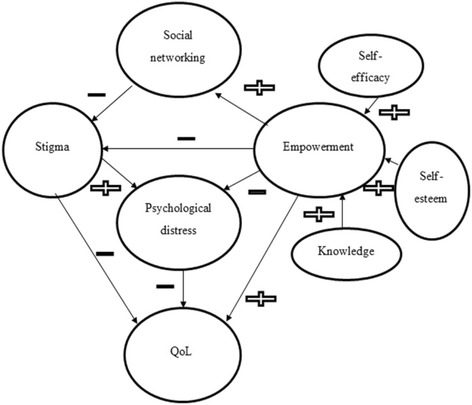
Model of PEI Impacts on Study Participants’ Outcomes Post-Intervention

Primary caregivers indicated they were able to redefine their relative’s problems and the situations that trigger stress, enabling them to tailor flexible and positive ways of coping. There are similarities between the findings of this study and those described by previous research [47, 48], which showed that an improvement in the family caregiver’s social life was associated with having an improved knowledge of their relatives’ illness. Consequently, there emerged an improvement in their sense of hope and empowerment to be socially active. This finding has much support in the literature, which indicates that improving family caregivers’ understanding of the illness might have assisted them in reappraising caregiving demands and better handling of maladaptive behaviour [38]. It is also important and interesting to note that the primary caregivers reported that their own social interaction was enhanced following the intervention. It may be that the intervention counteracts some of the negative effects of schizophrenia on their social life such as stigma, problematic behaviour of PDwS or emotional problems between participants. The significant improvement in the interviewed primary caregivers’ quality of life could be explained by the fact that the majority of them were female, and females may respond better to emotional and coping-related outcomes in PEIs, as illustrated in Fig. 2 [49].

The findings of this study have implications for clinical practice. From a policy perspective, the findings indicate that this less intrusive and less demanding and more cost-effective format of delivering intervention is as effective as face-to-face formats making it suitable for use in resource poor environments. Therefore, introducing helping resources for PDwS and primary caregivers support antipsychotic medication.

There are some limitations which could affect the transferability of the findings. Firstly, all interviews were conducted by the researcher who recruited participants into the study and implemented the intervention. This may be considered as both a strength and a limitation. It could be argued that the participants may be unlikely to disclose unfavourable opinions of the intervention to the individual that recruited them into the study. However, a number of participants did speak about aspects of the intervention they did not like and felt could be improved. Participants also reported some negative effects of the knowledge gained from the intervention, suggesting that they were able to speak about their experience openly. An alternative view is that participants felt more relaxed being interviewed by an individual with whom they had developed some rapport, enabling them to be more open in terms of their responses. It could also be considered that the inclusion of follow-up phone calls may represent a confounder in terms of understanding the impact specifically of the PEI and may limit the generalisability of the findings. Secondly; the research team was not able to recruit any male primary caregivers’ for interviews, whilst the quantitative study data suggests that males only represented 18% of the PCs recruited to the RCT, this lack of involvement may limit transferability of the findings.

The data from this study suggest that the implementation of this low-cost PEI into routine services should be possible. In terms of impact on the workload of the mental health professionals in Jordan it is suggested that they should allow PDwS and their PCs adequate time to discuss, understand and explore their experiences about schizophrenia, daily problems, coping with illness and antipsychotic medication. This may have a positive impact on satisfaction and adherence to treatment. These suggestions are supported by a growing body of evidence [50, 51]. It is encouraging that the findings of this study showed that the booklet format of delivering PEI, which has neither been tested in a controlled trial nor reported in the literature, demonstrated a similar effect on the mental condition as other forms of delivering PEIs.

## Conclusions

Individual understanding varied between PDwS and PCs reciving PEI and led to differences in the ways that they used knowledge gained from the PEI in everyday situations. These data support the importance of improving understanding of schizophrenia by PDwS and their PCs to enable them to benefit more fully from medication.
